# Identification of Differentially Expressed Genes in Breast Muscle and Skin Fat of Postnatal Pekin Duck

**DOI:** 10.1371/journal.pone.0107574

**Published:** 2014-09-29

**Authors:** Tieshan Xu, Lihong Gu, Kyle Michael Schachtschneider, Xiaolin Liu, Wei Huang, Ming Xie, Shuisheng Hou

**Affiliations:** 1 Institute of Animal Science (IAS), Chinese Academy of Agricultural Sciences (CAAS), Beijing, P.R. China; 2 Tropical Crops Genetic Resources Institute, Chinese Academy of Tropical Agricultural Science, Danzhuo, Hainan, P.R. China; 3 Department of Animal Science, University of Illinois, Urbana, Illinois, United States of America; 4 Shaanxi Key Laboratory of Molecular Biology for Agriculture, College of Animal Science and Technology, Northwest A&F University, Yangling, Shaanxi, P.R. China; University of Bonn, Germany

## Abstract

Lean-type Pekin duck is a commercial breed that has been obtained through long-term selection. Investigation of the differentially expressed genes in breast muscle and skin fat at different developmental stages will contribute to a comprehensive understanding of the potential mechanisms underlying the lean-type Pekin duck phenotype. In the present study, RNA-seq was performed on breast muscle and skin fat at 2-, 4- and 6-weeks of age. More than 89% of the annotated duck genes were covered by our RNA-seq dataset. Thousands of differentially expressed genes, including many important genes involved in the regulation of muscle development and fat deposition, were detected through comparison of the expression levels in the muscle and skin fat of the same time point, or the same tissue at different time points. KEGG pathway analysis showed that the differentially expressed genes clustered significantly in many muscle development and fat deposition related pathways such as MAPK signaling pathway, PPAR signaling pathway, Calcium signaling pathway, Fat digestion and absorption, and TGF-beta signaling pathway. The results presented here could provide a basis for further investigation of the mechanisms involved in muscle development and fat deposition in Pekin duck.

## Introduction

Pekin duck is a world-famous species for its fast growth, but its breast muscle yield is lower than that of other lean-type ducks [1]. Work carried out by the Chinese Academy of Agricultural Sciences since the 1990s has produced a new strain of lean-type Pekin duck with increased carcass skeletal muscle yield and decreased carcass fatness. This new strain of lean-type Pekin duck passed the national certification awarded by the Chinese State Variety Approval Committee of livestock and poultry in 2004. However, the potential mechanisms underlying increased muscle development and decreased fat deposition in lean-type Pekin ducks is unclear to date.

In birds, there are no significant changes in muscle fiber numbers during postnatal development [2], [3]. Instead, the postnatal muscle mass is increased by increasing the size of the muscle cells, a process referred to a hypertrophy that is controlled by both anabolic and catabolic mechanisms [4]. Among the complex hypertrophy regulating network, the insulin-like growth factor 1 (IGF1) signaling pathway plays a crucial role in promoting hypertrophy by activating tyrosine kinases, which activate phosphoinositide 3-kinase (PI3K)/Akt signaling [5], [6]. Conversely, forkhead box O (FOXO) family proteins inhibit hypertrophy of muscle fibers through suppression of the PI3K/Akt pathway [7]. In addition, adipose tissue mass is controlled by a balance of cell proliferation and an increase in fat cell size, known as hyperplasia and hypertrophy, respectively [8]. Multiple hormones and growth factors collaborate to regulate adipocyte differentiation and deposition [9]. Growth hormone (GH) has been shown to stimulate preadipocytes to undergo adipogenesis by priming the cells for the poliferative effect of IGF1 [10], a hormone which, in addition to insulin, is believed to be involved in adipocyte differentiation [11], [12]. In addition, it is believed that the expression of peroxisome proliferator-activated receptorα (PPARα) and CCAAT/enhancer binding protein α (C/EBPα) are important in the maintenance of the differentiated state of adipocytes [11]. Given the complexity involved in regulating skeletal muscle development and fat deposition, identification of the differentially expressed genes (DEGs) in duck breast muscle and skin fat is a critical first step to understanding the function of these genes.

In the past few years, next-generation high-throughput DNA sequencing techniques have provided fascinating opportunities in the life sciences and dramatically improved the efficiency and speed of gene discovery and DEGs exploration [13]. Previous studies have confirmed that the relatively short reads produced by Illumina sequencing can be effectively assembled and used for gene discovery and comparison of gene expression profiles [14], [15]. Identification of DEGs has been performed in many vertebrate species, including some bird species such as chicken [16], [17], goose [18], turkey [19] and zebra finch [20], [21]. Recently the duck (*Anas platyrhynchos*) genome sequence was finished [22] and the draft genome is now publicly available (http://www.ensembl.org/Anas_platyrhynchos/Info/Index). The duck genome will greatly improve the accuracy of duck RNA-seq analysis and will largely promote the identification and functional exploration of DEGs in duck.

Here we constructed six mRNA libraries. Three libraries from Pekin duck breast muscle at two-, four- and six-weeks of age (W2, W4 and W6, respectively), and three from Pekin duck skin fat at W2, W4 and W6. By high throughput RNA sequencing and subsequent bioinformatics analysis, we identified DEGs between Pekin duck breast muscle and skin fat samples. The results presented here could provide a basis for further functional investigation of DEGs between breast muscle and skin fat in Pekin duck.

## Materials and Methods

### Ethics Statement

All procedures of the present study were approved by the welfare committee of the Institute of Animal Science, Chinese Academy of Agricultural Sciences. All surgeries were performed according to recommendations proposed by the European Commission (1997), and all efforts were made to minimize the suffering of animals.

### Birds and Tissue Sample Collection

Thirty 1 day old Z5 Pekin duck (lean-type) full-sibs were selected randomly from the Pekin duck breeding farm at the Chinese Academy of Agricultural Science (Beijing, China), where they were raised under normal conditions. The dietary nutrient levels provided at different stages are listed in Table 1. Breast muscle and skin fat samples were collected from four healthy ducks selected at each time point (denoted as M2, M4, M6 and F2, F4, F6, respectively) for RNA isolation. The ducks were controlled for body weight by selecting ducks with an body weight within 100 g, 150 g, and 200 g of the average body weight of raised ducks at two-, four-, and six-weeks of age, respectively. All samples were snap-frozen in liquid nitrogen and stored at −80°C.

**Table 1:** The dietary nutrient levels for Pekin duck at different stages.

Nutritional items	1 week∽2 weeks	3weeks∽5 weeks	6 weeks∽7 weeks
**Apparent metabolizable energy, MJ/kg**	12.14	12.14	12.35
**Crude protein, %**	20.0	17.5	16.0
**Calcium, %**	0.90	0.85	0.80
**Total phosphorus, %**	0.65	0.60	0.55
**Na, %**	0.15	0.15	0.15
**Cl, %**	0.12	0.12	0.12
**Lysine, %**	1.10	0.85	0.65
**Methionine, %**	0.45	0.40	0.35
**Methionine + Cystine, %**	0.80	0.70	0.60
**Threonine, %**	0.75	0.60	0.55
**Tryptophan, %**	0.22	0.19	0.16
**Arginine, %**	0.95	0.85	0.70
**Isoleucine, %**	0.72	0.57	0.45
**Vitamin A, IU/kg**	4000	3000	2500
**Vitamin D3, IU/kg**	2000	2000	2000
**Vitamin E, IU/kg**	20	20	10
**Vitamin K3, mg/kg**	2.0	2.0	2.0
**Vitamin B1, mg/kg**	2.0	1.5	1.5
**Vitamin B2, mg/kg**	10	10	10
**Niacin, mg/kg**	50	50	50
**Pantothenic acid**, mg/kg	20	10	10
**Vitamin B6, mg/kg**	4.0	3.0	3.0
**Vitamin B12, mg/kg**	0.02	0.02	0.02
**Biotin, mg/kg**	0.15	0.15	0.15
**Folic acid, mg/kg**	1.0	1.0	1.0
**Choline, mg/kg**	1000	1000	1000
**Cu, mg/kg**	8.0	8.0	8.0
**Fe, mg/kg**	60	60	60
**Mn, mg/kg**	100	100	100
**Zn, mg/kg**	60	60	60
**Selenium, mg/kg**	0.30	0.30	0.20
**I, mg/kg**	0.40	0.40	0.30

### RNA isolation, Library Preparation and Illumina Sequencing

Tissue samples were sent to BGI for RNA isolation, library preparation and Illumina sequencing. Breifly, total RNA was isolated from all breast muscle and skin fat samples using the RNAiso plus kit (Takara) following the manufacturer's instructions. The total RNA samples were mixed in equimolar ratio to generate an RNA pool for each tissue and time point (M2, M4, M6 and F2, F4, F6). The RNA quality was analyzed by 1.0% agarose gel electrophoresis and spectrophotometric absorption at 260 nm in a Nanodrop ND-1000 Spectrophotometer. All RNA samples were treated with DNase I recombinant (Roche). The mRNA was separated from 6 mg of total RNA using oligo (dT) magnetic beads and fragmented into short fragments using the fragmentation buffer. First strand cDNA was synthesized from the short mRNA fragments using a random hexamer-primer, purified and dissolved in EB buffer for end repair and single nucleotide A (adenine) addition. Subsequently, sequencing adapters were ligated to the 5′ and 3′ ends of the fragments. The fragments were amplified by PCR amplification and products with 5′and 3′ adapters were purified by agarose gel electrophoresis. Agilent 2100 Bioanaylzer and ABI StepOnePlus Real-Time PCR System were used in quantification and qualification of the sample libraries. Finally, the libraries were sequenced on Illumina sequencing platform (HiSeq 2000).

### Quality control and filtering of raw reads

Quality control was first performed on the primary sequencing data produced by Illumina HiSeq 2000. We then carried out adapter trimming and filtering of the initial reads to decrease the data noise. Specifically, the reads were trimmed and filtered to remove: i) adapter contamination, ii) reads in which unknown bases were more than 5% of the read and iii) low quality reads(Q score <20). The filtered reads (high-quality reads) were used for downstream analysis.

### Alignment of high-quality reads to reference

The high-quality reads were aligned to the duck reference transcriptome using SOAPaligner/SOAP2 [23] with no more than 2 mismatches. Basic alignment statistics were performed, and the distribution of the reads on the reference transcriptome was determined to evaluate the randomness. Subsequent gene coverage was calculated to determine the percentage of a gene covered by reads.

### Analysis of DEGs

DEGs between samples were determined based on the reads per kilobase per million reads (RPKM) method [24]. The RPKM value of for each gene with a minimum RPKM value of 0.001 was compared between each tissue and time point using the method described by Audic and Claverie [25]. The false discovery rate (FDR) [26], which was used to determine the threshold of the P value in multiple tests and analyses, was set at less than 0.001 to judge the significance of gene expression differences.

### Gene ontology (GO) and pathway enrichment analysis of DEGs

We firstly mapped all DEGs to GO terms in the database (http://www.geneontology.org/) and calculated gene numbers for every GO term. Significantly enriched GO terms were determined using hypergeometric distribution based on ‘GO::TermFinder' (http://smd.princeton.edu/help/GO-TermFinder/GO_TermFinder_help.shtml). To further understand the biological functions of the DEGs, KEGG [27] was used to perform pathway enrichment analysis.

### Real time quantitative polymerase chain reaction (qRT-PCR)

Real time quantitative PCR (qRT-PCR) was performed using the SYBR PrimeScript RT-PCR Kit (TaKaRa) with SYBR Green dye to validate specific gene transcription, RNA-Seq data and variations in gene expression among individuals. The RNA used for qRT-PCR was prepared in the same manner as the total RNA extraction and DNase I treatment described above. A reference gene (β-actin) was used as a control for detecting the expression levels of these genes. The primer pairs used for qRT-PCR are listed in Table 2 and the RPKM values of the selected 18 genes are present in Table 3. The qRT-PCR reactions were carried out with an iCycler IQ5 Multicolor Real-Time PCR Detection System (Bio-Rad). The qRT-PCR reaction contained 1 µL of cDNA template, 12.5 µL of SYBR Premix ExTaq, 9.5 µL of sterile water, and 1 µL of each gene-specific primer. Thermal cycling parameters were 1 cycle at 95°C for 2 min, 40 cycles of 95°C for 15 s, and 60°C for 34 s. Dissociation curve analysis was done after each real time reaction to ensure that there was only one product. The qRT-PCR analysis of each sample was done in triplicate.

**Table 2:** Genes used for qRT-PCR and their primers.

Gene symbol	Description	Primers sequence
**EEF1A1**	*	F: CACCGAGCCACCTTACAG
		R: AGGATGCAGTCCAGAGCC
**COL1A2**	*	F: GCGGTTTCTACTGGATTG
		R: TTCAAACTGACTGCCACC
**RPL4**	*	F: TGAGACTTGCTCCTGGTG
		R: AGGTCCGTATTGGTCATC
**RPSA**	*	F: CAGCCCGTGCTATTGTGG
		R: CCGCCTGGATCTGATTTG
**RPS24**	*	F: AAGCAGCGGAAGGAACG
		R:AACCAGGATTTGACTGACATAG
**EEF1B2**	$	F: ACGGTCCTGCTGATGTTG
		R: GCCAGACGTTCTTCCCTC
**SCD**	$	F: CAGCGGAAATACTACAAGC
		R:TGAGCAGCACTGTTCACTAG
**RPL5**	$	F: TCCCTCATAGTACCAAGC
		R: GCATCTTCATCTTCCTCC
**RPS8**	$	F: CAGTGAGGGTTCGTGGTG
		R: TCACCAGGGTCTTCGTCC
**RPS2**	$	F: GGTCTGGGCGTGAAATGC
		R: TGTGGGGCTTGCCGATC
**IGF1R**	#	F: ATGTGGTTCGGTTGCTTG
		R: AACTTGTTGGCGTTGAGG
**IGF1**	#	F: GTTGATGCTCTTCAGTTCG
		R: CCTCCTCAGGTCACAACTC
**TGIF1**	#	F: CAACGCCTTCACAGACAG
		R: TCCTGGTTGAGGTCTGGT
**FOXO6**	#	F: ATGTGGTTCGGTTGCTTG
		R: GTCGCTGTCCATAAAGTCATTC
**MSTN**	#	F: TTACAGACACACCGAAACG
		R: TCTCCAGAGCAGTAATCGG
**TGFβ3**	#	F: TTTCTCGGTCTTGTTGCC
		R: TCTCATTCCTTGCCTTCC
**TGFβ1**	#	F:ATGAGTATTGGGCCAAAGAG
		R:CGTTGAACACGAAGAAGATG
**FOXO3**	#	F:CGTATGGTTCCAAAGGTTCG
		R:CAGCGTCTGGTTGTTGTAATGG

Note: * used for validation of RNA-seq data, $ used for validation of genetic variation among individuals, # used for validation of important DEGs involvedin muscle development and fat deposition.

**Table 3:** RNA-seq RPKM values of 18 genes selected.

Gene symbol	F2	F4	F6	M2	M4	M6
**EEF1A1**	9352.00	9543.22	9518.95	2520.50	1673.78	1806.92
**COL1A2**	5316.93	2411.59	1877.42	920.84	821.97	176.53
**RPL4**	2815.86	2837.10	1953.63	2261.97	1412.46	1059.19
**RPSA**	2513.99	2540.59	1547.16	2294.97	1406.68	781.25
**RPS24**	5031.61	4926.67	3764.16	2956.06	2371.45	1706.44
**EEF1B2**	3747.69	3700.62	3945.39	5036.90	3813.65	2813.71
**SCD**	2219.44	3378.68	1467.31	5.77	1.44	3.58
**RPL5**	2356.04	2487.96	1760.44	1783.96	1187.15	899.01
**RPS8**	4425.85	5244.55	3358.78	4622.44	2974.36	2333.82
**RPS2**	2175.67	2167.37	1204.14	3593.80	2419.23	2160.93
**IGF1R**	6.10	6.34	4.36	1.14	1.39	1.42
**IGF1**	1.55	1.61	0.51	0.00	2.01	2.16
**TGIF1**	13.80	14.83	6.59	6.35	3.45	0.00
**FOXO6**	16.25	11.94	11.45	5.01	0.67	0.11
**MSTN**	1.27	1.04	0.41	107.72	91.42	56.69
**TGFβ3**	140.71	173.34	88.05	364.85	138.30	89.99
**TGFβ1**	95.62	82.70	90.08	43.74	26.22	16.06
**FOXO3**	20.38	12.94	44.94	34.86	16.85	16.46

The relative gene expression level was determined by the comparative cycle threshold (C_T_) method [28]. The ΔC_T_ value was calculated by subtracting the target C_T_ of each sample from the β-actin C_T_ value.

### Data deposition

Data described in this study is available in the NIH Short Read Archive (SRA) under accession number SRX393261.

## Results and Discussion

### Sequencing, quality control and filtering of initial reads

RNA-seq data were obtained from three breast muscle samples (M2, M4, and M6) and three skin fat samples (F2, F4, and F6). Around 31∼47 million initial reads were obtained from each sample, with a total of 228 million initial reads generated. After quality control and adapter removal, 29∼45 million high-quality reads for each sample and a total of 218 million high-quality reads were available for further analysis (Table S1). Overall, ∼95.76% of the raw reads were classified as high-quality reads. This indicates that the data are sufficient in sequencing depth and read quality and that the data are well suitable for the analysis of DEGs in breast muscles and skin fats of Pekin duck.

### Alignment of high-quality reads against the duck reference transcriptome

After quality control and filtering of initial reads, we aligned the high-quality reads obtained above to the reference transcriptome (ftp://ftp.ensembl.org/pub/pre/fasta/cdna/anas_platyrhynchos/Anas_platyrhynchos.duck_1.cdna.fa.gz).

The statistical results for read alignment against the duck transcriptome are summarized in Table 4, 5, 6, 7, 8, 9, 10. For the total dataset (combined data from all six samples), 44.31% of the reads aligned to the duck transcriptome, with 42.27% aligning uniquely. The alignment rate for the six samples varied, ranging from 38.67% for F4 to 47.26% for M4. In this study, the percent uniquely aligned reads is lower than in studies performed by Eizirik et al. and Djebali et al. in humans [29], [30], and slightly lower than the results of Li et al. in chickens [31]. This may be due to the fact that the duck genome is only a draft, and requires more work to improve it to the level of chicken or human.

**Table 4:** Read alignment statistics against the duck transcriptome of total.

Map to Gene	Number	Percentage
**Total reads**	218,609,846	100.00%
**Total mapped reads**	96,859,805	44.31%
**Perfect matched reads**	64,873,987	29.68%
**Reads with <5 mismatched bases**	31,985,818	14.63%
**Unique matched reads**	92,412,723	42.27%
**Multiple matched Reads**	4,447,082	2.03%
**Total unmapped reads**	121,750,041	55.69%

**Table 5:** Read alignment statistics against the duck transcriptome of F2.

Map to Gene	Number	Percentage
**Total reads**	40,441,610	100.00%
**Total mapped reads**	18,231,246	45.08%
**Perfect matched reads**	12,053,481	29.80%
**Reads with <5 mismatched bases**	6,177,765	15.28%
**Unique matched reads**	17,474,453	43.21%
**Multiple matched Reads**	756,793	1.87%
**Total unmapped reads**	22,210,364	54.92%

**Table 6:** Read alignment statistics against the duck transcriptome of F4.

Map to Gene	Number	Percentage
**Total reads**	39,110,110	100.00%
**Total mapped reads**	15,123,342	38.67%
**Perfect matched reads**	9,510,860	24.32%
**Reads with <5 mismatched bases**	5,612,482	14.35%
**Unique matched reads**	13,898,212	35.54%
**Multiple matched Reads**	1,225,130	3.13%
**Total unmapped reads**	23,986,768	61.33%

**Table 7:** Read alignment statistics against the duck transcriptome of F6.

Map to Gene	Number	Percentage
**Total reads**	45,427,820	100.00%
**Total mapped reads**	20,392,268	44.89%
**Perfect matched reads**	13,373,732	29.44%
**Reads with <5 mismatched bases**	7,018,536	15.45%
**Unique matched reads**	19,737,726	43.45%
**Multiple matched Reads**	654,542	1.44%
**Total unmapped reads**	25,035,552	55.11%

**Table 8:** Read alignment statistics against the duck transcriptome of M2.

Map to Gene	Number	Percentage
**Total reads**	30,954,282	100.00%
**Total mapped reads**	14,015,868	45.28%
**Perfect matched reads**	9,711,269	31.37%
**Reads with <5 mismatched bases**	4,304,599	13.91%
**Unique matched reads**	13,416,376	43.34%
**Multiple matched Reads**	599,492	1.94%
**Total unmapped reads**	16,938,414	54.72%

**Table 9:** Read alignment statistics against the duck transcriptome of M4.

Map to Gene	Number	Percentage
**Total reads**	33,230,656	100.00%
**Total mapped reads**	15,704,060	47.26%
**Perfect matched reads**	10,988,612	33.07%
**Reads with <5 mismatched bases**	4,715,448	14.19%
**Unique matched reads**	15,011,755	45.17%
**Multiple matched Reads**	692,305	2.08%
**Total unmapped reads**	17,526,596	52.74%

**Table 10:** Read alignment statistics against the duck transcriptome of M6.

Map to Gene	Number	Percentage
**Total reads**	29,445,368	100.00%
**Total mapped reads**	13,393,021	45.48%
**Perfect matched reads**	9,236,033	31.37%
**Reads with <5 mismatched bases**	4,156,988	14.12%
**Unique matched reads**	12,874,201	43.72%
**Multiple matched Reads**	518,820	1.76%
**Total unmapped reads**	16,052,347	54.52%

**Figure 1:**
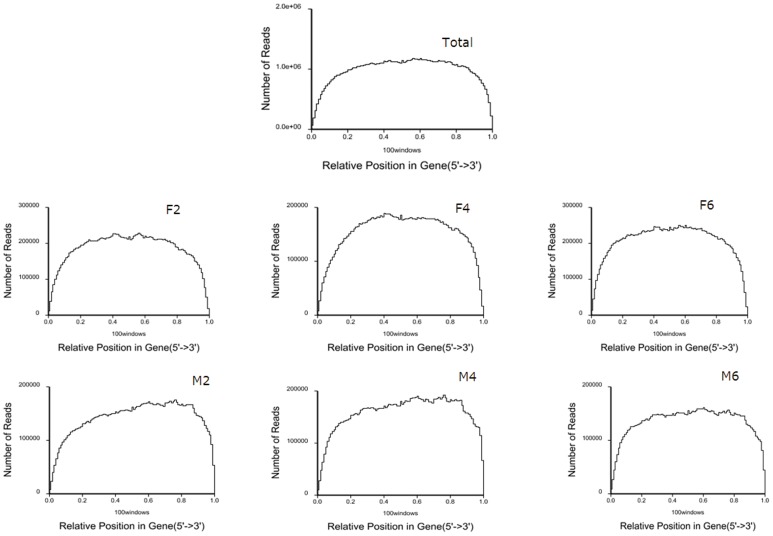
Random distribution of reads across genes. Note: Figure 1A–1G represent the reads across genes from the total RNA-seq dataset (All), reads from the skin fat sample at 2-, 4- and 6-weeks of age (F2, F4 and F6); reads from the breast muscle sample at 2-, 4- and 6-weeks of age (M2, M4 and M6) respectively,

For excellent RNA-seq data, the reads should randomly distribute along the transcriptome [32]. In this study, we first standardized a read location on a gene to a relative position and then counted the number of reads in each relative position to evaluate read randomness. The read distribution along the duck transcriptome is shown in Fig. 1. Overall, most reads were aligned at the body of the genes for each of the six sample datasets, indicating that the mRNA used for sequencing in this study was randomly broken into segments.

**Figure 2:**
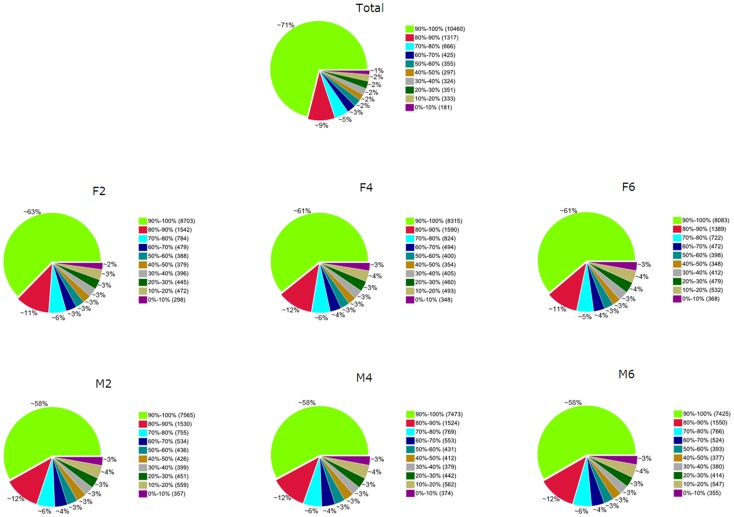
Coverage statistics of annotated duck genes by the current RNA-seq data. Note: Figure 2A-2G represent percent of each gene covered by the total RNA-seq dataset coverage (All), the skin fat samples at 2-, 4- and 6-weeks of age (F2, F4 and F6), and the breast muscle samples at 2-, 4- and 6-weeks of age (M2, M4 and M6) respectively.

The average expression of each mapped gene was calculated using the RPKM method [24]. For the total dataset, 14,709 genes, representing 89.4% of the annotated duck genes expressed in our dataset. The number of expressed genes ranged from 12,731 (M6) to 13,886 (F2; Table 11). Furthermore, analysis of the coverage of each gene was performed to determine if a gene was fully covered (>90%) by the reads. Around 71% of the annotated duck genes were fully covered by reads for the total dataset, and 58% to 63% were fully covered for each sample (Fig. 2).

**Table 11:** Number of expressed genes in each sample.

Sample	Number of expressed genes
**F2**	13,886
**F4**	13,683
**F6**	13,203
**M2**	13,012
**M4**	12,929
**M6**	12,731
**Total (combine the data above)**	14,709

**Figure 3:**
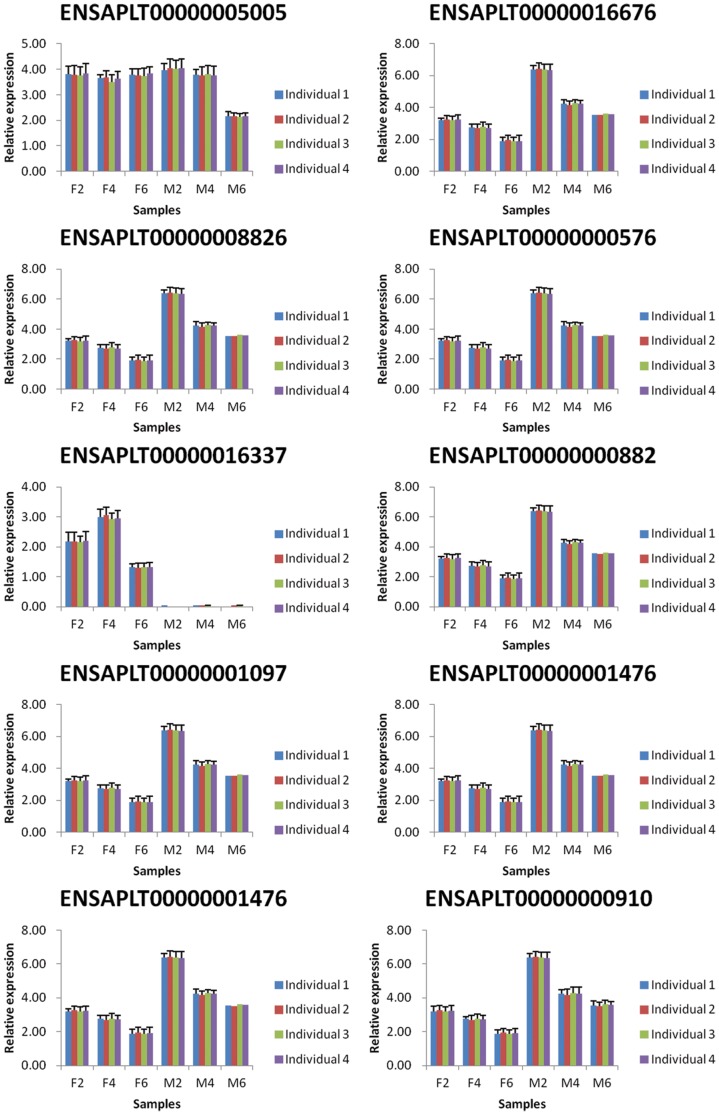
Validation of genetic variation among individuals. Note: The validation of genetic variation among individuals was performed by comparing of the relative expression of ten randomly selected genes within each tissue type and time point using qRT-PCR.

**Figure 4:**
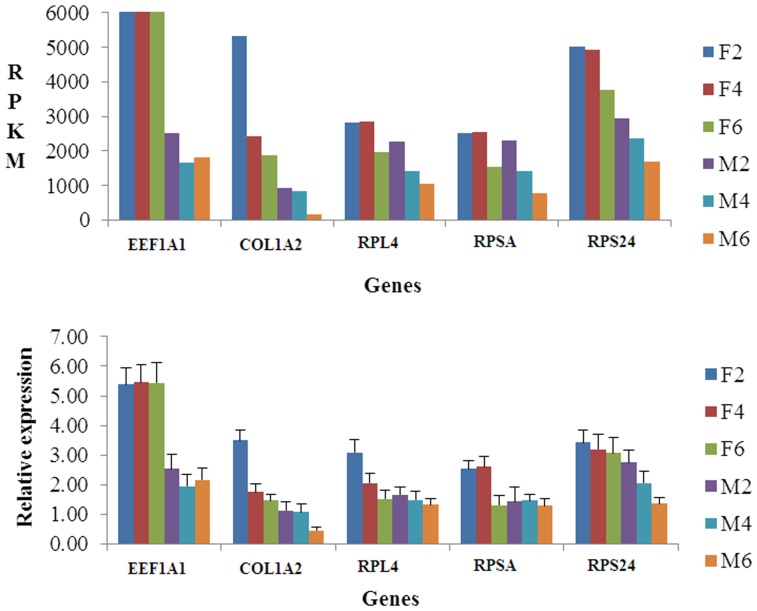
Validation of the accuracy of RNA-seq data. Note: The validation of the accuracy of RNA-seq data was carried out by comparing of RPKM and the relative expression of five randomly selected genes using qRT-PCR for each tissue and time point.

Because genetic variation between individuals can be extensive and inevitable, we selected 12 ducklings from a group of 30 full-sibs raised under the same conditions in order to reduce the variation observed within groups. In addition, to determine the variation in gene expression among samples within the groups studied, we performed real-time quantitative PCR on 10 randomly selected genes (Fig. 3). The results showed no significant differences among samples within each group, indicating genetic variation among individuals is not significant. In addition, to verify the accuracy of the RNA-seq data, we compared the RPKM and relative expressions of another five randomly selected genes using qRT-PCR (Fig. 4). The expression trends obtained by using qRT- PCR were highly consistent with the RPKMs of the five genes, suggesting the RNA-seq data are accurate.

### Analysis of DEGs

**Figure 5:**
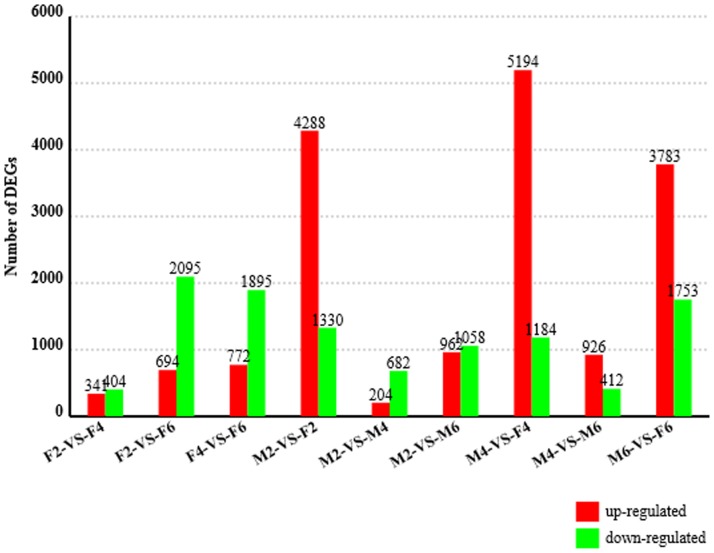
Differentially expressed genes between each sample pair. Note: The number of differentially expressed genes found in each sample pair, expressed as the number of up-regulated and down-regulated genes. The first sample in each comparison is considered the control sample. For example, in the F2-VS-F4 comparison, 341 genes are up-regulated in F4 compared to F2.

High-throughput sequencing technology is rapidly becoming the standard method for measuring RNA expression levels [24]. One of the main goals of these experiments is to identify DEGs. Adipocytes share a common mesenchymal cell origin with skeletal muscle cells [33], [34]. However unlike muscle cells, a lack of exposure to certain signaling factors [35] triggers the differentiation of cells into fat cells. Therefore, investigating the differences in gene expression levels between breast muscles and skin fat at multiple time points of development will be very helpful in exploring the molecular mechanisms underlining the lean-type duck phenotype. Only genes that met the following criteria were treated as DEGs: expression between two conditions differs by more than 1 fold change and FDR <0.001. In total, hundreds to thousands of genes were differentially expressed between the various tissues and time points. Generally, the number of DEGs between breast muscle and skin fat were much larger than between time points within tissues (Fig. 5, Table S2). More DEGs showed higher expression in skin fat than in breast muscle, suggesting that gene transcription in skin fat is more active than that in breast muscle. When comparing DEGs in F6 with that of F4 and F2, 694 and 772 genes showed higher expression, while 2095 and 1895 genes showed lower expression, respectively. The large number of DEGs between tissues and time points indicate that there are huge differences in gene transcription required for breast muscle and skin fat development. According to the RPKM values of DEGs at different tissues and time points, the DEGs could be divided into three expression patterns: those whose expression levels increase in skin fat and decrease in breast muscle with duck growth, those whose expression levels decrease in skin fat and increase in breast muscle with duck growth, and those whose expression levels did not follow any obvious pattern. Given that the breast muscle growth rate increases from 2 to 6 weeks of age, the first type of DEGs may be inhibitors of breast muscle development, while the second type may be promoters.

### Gene ontology (GO) and KEGG pathway analysis of DEGs

The GO project is a collaborative effort to develop and use ontologies to support biologically meaningful annotation of genes and their products [36]. In this study, we performed GO analysis to describe the properties of DEGs in duck breast muscle and skin fat and screened for significantly enriched GO terms (P<0.05) for each sample pair (Table S3). For cellular components, DEGs in different sample pairs were significantly enriched (P<0.05) into various GO terms, include contractile fiber between F2 and F4, F2 and F6, and F4 and F6; membrane and membrane part between M2 and F2; cytoplasmic part and cytoplasm between M6 and F6, and plasma membrane between M2 and M6. For biological processes, the DEGs between F2 and F4 and between F2 and F6 were significantly enriched into muscle development related terms (muscle cell development, striated muscle cell development, muscle fiber development, muscle system process, muscle structure development, striated muscle cell differentiation and muscle contraction). In addition, the DEGs between F4 and F6, M2 and F2 and M2 and M6 were extremely significantly enriched (P<0.01) multicellular organismal processes and single-multicellular organism processes.

KEGG is a "computer representation" of the biological system [37] and the KEGG database can be utilized for modeling and simulation, browsing and retrieval of data. In this work, we performed KEGG pathway analysis of DEGs to determine the significantly enriched pathways (P<0.05) for each sample pair (Table S4). The DEGs were significantly enriched into some pathways of muscle development or fat deposition. For example, the DEGs between F2 and F4, F2 and F6, F4 and F6 and M2 and F2 were enriched significantly enriched into the MAPK signaling pathway, a major regulator of skeletal muscle development [38]. The DEGs in all sample pairs excepting between F2 and F4 and between F2 and F6 were significantly enriched into PPAR signaling pathway, which is able to increase recruitment, proliferation and differentiation of preadipocytes leading ultimately to improved adipose tissue [39]. The DEGs between M2 and F2 and M4 and F4 were significantly enriched into wnt signaling pathway, which has been implied to be a molecular switch that governs adipogenesis [40]. Other pathways involved in muscle development and fat deposition that were significantly enriched by DEGs in one or more sample pairs include fat digestion and absorption, phosphatidylinositol signaling system, GnRH signaling pathway, fatty acid metabolism and p53 signaling pathway. A number of pathways enriched into by DEGs are involved in muscle development and fat deposition, suggesting that muscle development and fat deposition are major events that require expression of different genes.

### Important DEGs involved in muscle development and fat deposition of duck

The formation of a specific phenotype is a result of the interaction of a certain genotype with the environment, and environmental factors exert their influence on phenotype through genetic or epigenetic mechanisms [31]. The genetic effects perform a natural role in this process. In this study, a number of important genes for muscle development and fat deposition were differentially expressed between various tissues and time points. They include insulin-like growth factor 1 receptor (IGF1R), IGF1, myostatin (MSTN), transforming growth factor beta 3 (TGFβ3), transforming growth factor beta-induced 1 (TGFβ1), TGFβ-induced factor homeobox 1 (TGIF1), forkhead box O6 (FOXO6), forkhead box O3 (FOXO3) and the members of the PPAR signaling pathway and the MAPK signaling pathway, which are described in detail below.

**Figure 6:**
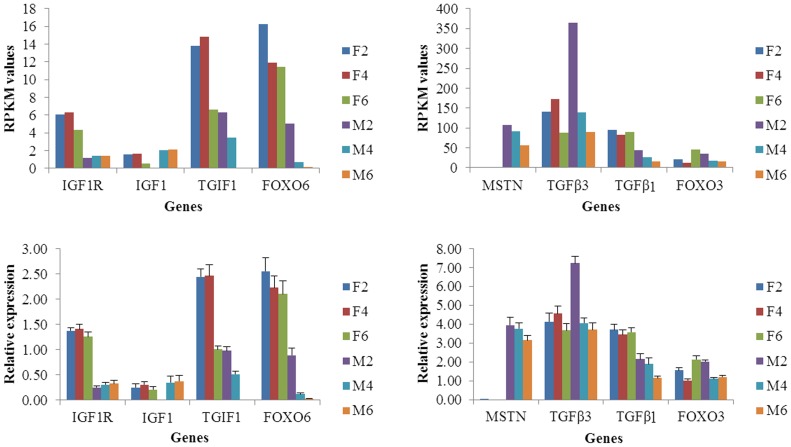
Validation of expression levels of important genes involved in muscle development and fat deposition. Note: The validation of important genes was performed by comparing of RPKM and relative expression using qRT- PCR for each tissue and time point.

To validate these genes, we performed qRT-PCR for each gene mentioned above and compared the expression level with the RPKM value of each gene (Fig. 6, Table 3). It has been reported that IGF1 can stimulate the development of muscle mass by increasing protein synthesis while decreasing proteolysis and myogenesis [41]. In this report, the expressions of IGF1 and IGF1R increased in breast muscles with duck growth, suggesting the breast muscle development increases from 2 to 6 weeks of age. The expressions of MSTN, TGFβ3, TGFβI, TGIF1, FOXO6 and FOXO3 were all decreased in the breast muscle with increasing age, but fluctuated in the skin fat samples. MSTN is a member of the transforming growth factor β superfamily of secreted growth factors that negatively regulates skeletal muscle size [42]. In mature adult muscle, TGF-β negatively affects skeletal muscle regeneration by inhibiting satellite cell proliferation, myofiber fusion, and expression of some muscle-specific genes [43] and TGF-β1 induces the transformation of myogenic cells into fibrotic cells after injury [44]. In addition, inhibition of FoxO transcriptional activity by expression of a dominant negative (DN) FOXO allele prevents at least half of disuse-induced muscle fiber atrophy in vivo [45], [46], and muscle-specific over expression of FOXO1 or FOXO3a is sufficient to cause skeletal muscle atrophy in vivo [47], [48]. A number of inhibitors for muscle mass growth showed a trend for decreased expression over time in this study, indicating that the development of breast muscle increases with the age of the duck.

### DEGs in the MAPK signaling pathway

Notably, the MAPK signaling pathway contained many DEGs in the breast muscle and skin fat sample pairs (M2-VS-F2, M4-VS-F4 and M6-VS-F6; Table S4). We therefore summarized the DEGs in the pathway in Table S5.

The MAPK pathway is a chain of proteins in the cell that communicates signals from a receptor on the surface of the cell to the DNA in the nucleus of the cell. The MAPKs are responsible for a variety of processes including the transcriptional activation of cytokines, chemokines and other inflammatory mediators [49]. The p38 MAPK signaling pathway, one module of the MAPK protein family, is a major regulator of skeletal muscle development [38]. In this study, we observed 214 DEGs in the MAPK signaling pathway between breast muscle and skin fat sample pairs. Some members of this pathway, which are crucial for skeletal development, were identified as highly expressed in breast muscle compared to skin fat. For example, it is reported that serum response factor (SRF) is required for satellite cell-mediated hypertrophic muscle growth [50]. The expression of SRF in breast muscle was significantly higher than in skin fat in this study. Also, the expression of voltage-dependent calcium channel gamma subunit 1 (CACNG1), a regulator of skeletal muscle strength [51], was enriched in breast muscle (RPKM >500) compared to skin fat (RPKM <7). In addition, it has been implied that fibroblast growth factor (FGF) plays a prominent role in regulating muscle hypertrophy [52] and inducing hypertrophy and angiogenesis in hibernating myocardium [53]. In this work, many FGFs, such as FGF13, FGF1, FGF6, FGF4 and FGF10 were identified as DEGs, and most of them were highly expressed in breast muscle compared to skin fat. The results above indicate that the phenotypic formation of lean-type Pekin duck may be due to differences in gene expression between breast muscle and skin fat.

In summary, we identified and analyzed DEGs in breast muscle and skin fat of Pekin duck using RNA-seq data. To our knowledge, this is the first time that RNA-seq analysis with duck reference genome has been done in ducks. The current work provides important basic information for further comprehensive investigation of the mechanisms involved in muscle development and fat deposition of duck.

## Supporting Information

Table S1 **Statistical results for filtering of initial reads.**
(DOCX)

Table S2 **Differentially expressed genes between sample pairs.**
(XLSX)

Table S3 **Terms from the Gene Ontology with p-value lower than 0.05.**
(XLSX)

Table S4 **Significant pathways (P<0.05) from KEGG pathway analysis of DEGs.**
(XLSX)

Table S5 **Differentially expressed genes in MAPK pathway and their RPKM values in various tissues and time points.**
(XLSX)
