# Supplementary material for: Identification of Differentially Expressed Genes in Breast Muscle and Skin Fat of Postnatal Pekin Duck
Source: PLoS One. 2014 Sep 29;9(9):e107574. doi: 10.1371/journal.pone.0107574 (PMC4180276; doi:10.1371/journal.pone.0107574)
Supplement: Table S1 — Statistical results for filtering of initial reads. (DOCX) [file pone.0107574.s001.docx]

Table S1 The statistical results of filtering initial reads

| Samples | Items | Before filter | | | After filter | | |
| --- | --- | --- | --- | --- | --- | --- | --- |
|  |  | Total | Reads1.fq | Reads2.fq | Total | Reads1.fq | Reads2.fq |
| Total | Total reads | 228291264 | 114145632 | 114145632 | 218609846 | 109304923 | 109304923 |
|  | Accumulated length (nt) | 22829126400 | 11414563200 | 11414563200 | 21860984600 | 10930492300 | 10930492300 |
|  | Q20 number | 21723855572 | 10994944237 | 10728911335 | 21070207129 | 10616405413 | 10453801716 |
|  | Q20 percentage | 95.16% | 96.32% | 93.99% | 96.38% | 97.13% | 95.64% |
|  | GC number | 11639202838 | 5819124336 | 5820078502 | 11139889132 | 5569199507 | 5570689625 |
|  | GC percentage | 50.98% | 50.98% | 50.99% | 50.96% | 50.95% | 50.96% |
|  | N number | 10355057 | 304184 | 10050873 | 2972300 | 113243 | 2859057 |
|  | N percentage | 0.05% | 0.00% | 0.09% | 0.01% | 0.00% | 0.03% |
| F2 | Total reads | 41613298 | 20806649 | 20806649 | 40441610 | 20220805 | 20220805 |
|  | Accumulated length (nt) | 4161329800 | 2080664900 | 2080664900 | 4044161000 | 2022080500 | 2022080500 |
|  | Q20 number | 3945066571 | 2005688199 | 1939378372 | 3852588945 | 1953896641 | 1898692304 |
|  | Q20 percentage | 94.80% | 96.40% | 93.21% | 95.26% | 96.63% | 93.90% |
|  | GC number | 2132970552 | 1066312348 | 1066658204 | 2073708230 | 1036254522 | 1037453708 |
|  | GC percentage | 51.26% | 51.25% | 51.27% | 51.28% | 51.25% | 51.31% |
|  | N number | 3408983 | 59089 | 3349894 | 1039418 | 26502 | 1012916 |
|  | N percentage | 0.08% | 0.00% | 0.16% | 0.03% | 0.00% | 0.05% |
| F4 | Total reads | 40253330 | 20126665 | 20126665 | 39110110 | 19555055 | 19555055 |
|  | Accumulated length (nt) | 4025333000 | 2012666500 | 2012666500 | 3911011000 | 1955505500 | 1955505500 |
|  | Q20 number | 3820764352 | 1941419414 | 1879344938 | 3729699781 | 1890716974 | 1838982807 |
|  | Q20 percentage | 94.92% | 96.46% | 93.38% | 95.36% | 96.69% | 94.04% |
|  | GC number | 2110149080 | 1054869953 | 1055279127 | 2051610341 | 1025214837 | 1026395504 |
|  | GC percentage | 52.42% | 52.41% | 52.43% | 52.46% | 52.43% | 52.49% |
|  | N number | 3023011 | 57789 | 2965222 | 835668 | 25612 | 810056 |
|  | N percentage | 0.08% | 0.00% | 0.15% | 0.02% | 0.00% | 0.04% |
| F6 | Total reads | 47036174 | 23518087 | 23518087 | 45427820 | 22713910 | 22713910 |
|  | Accumulated length (nt) | 4703617400 | 2351808700 | 2351808700 | 4542782000 | 2271391000 | 2271391000 |
|  | Q20 number | 4461297553 | 2267401832 | 2193895721 | 4329818016 | 2195143250 | 2134674766 |
|  | Q20 percentage | 94.85% | 96.41% | 93.29% | 95.31% | 96.64% | 93.98% |
|  | GC number | 2379569495 | 1189571379 | 1189998116 | 2296934760 | 1147871677 | 1149063083 |
|  | GC percentage | 50.59% | 50.58% | 50.60% | 50.56% | 50.54% | 50.59% |
|  | N number | 3582059 | 68684 | 3513375 | 1055304 | 29811 | 1025493 |
|  | N percentage | 0.08% | 0.00% | 0.15% | 0.02% | 0.00% | 0.05% |
| M2 | Total reads | 32708780 | 16354390 | 16354390 | 30954282 | 15477141 | 15477141 |
|  | Accumulated length (nt) | 3270878000 | 1635439000 | 1635439000 | 3095428200 | 1547714100 | 1547714100 |
|  | Q20 number | 3130424945 | 1573739488 | 1556685457 | 3025471356 | 1511876548 | 1513594808 |
|  | Q20 percentage | 95.71% | 96.23% | 95.18% | 97.74% | 97.68% | 97.80% |
|  | GC number | 1642696741 | 821527979 | 821168762 | 1550927162 | 775747962 | 775179200 |
|  | GC percentage | 50.22% | 50.23% | 50.21% | 50.10% | 50.12% | 50.09% |
|  | N number | 110149 | 38249 | 71900 | 13527 | 10249 | 3278 |
|  | N percentage | 0.00% | 0.00% | 0.00% | 0.00% | 0.00% | 0.00% |
| M4 | Total reads | 35780424 | 17890212 | 17890212 | 3323065 | 16615328 | 16615328 |
|  | Accumulated length (nt) | 3578042400 | 1789021200 | 1789021200 | 3323065600 | 1661532800 | 1661532800 |
|  | Q20 number | 3392698740 | 1712979069 | 1679719671 | 3248402119 | 1623278120 | 1625123999 |
|  | Q20 percentage | 94.82% | 95.75% | 93.89% | 97.75% | 97.70% | 97.81% |
|  | GC number | 1811024662 | 905112060 | 905912602 | 1680213704 | 840471305 | 839742399 |
|  | GC percentage | 50.61% | 50.59% | 50.64% | 50.56% | 50.58% | 50.54% |
|  | N number | 123551 | 42887 | 80664 | 14961 | 11148 | 3813 |
|  | N percentage | 0.00% | 0.00% | 0.00% | 0.00% | 0.00% | 0.00% |
| M6 | Total reads | 30899258 | 15449629 | 15449629 | 29445368 | 14722684 | 14722684 |
|  | Accumulated length (nt) | 3089925800 | 1544962900 | 1544962900 | 2944536800 | 1472268400 | 1472268400 |
|  | Q20 number | 2973603411 | 1493716235 | 1479887176 | 2884226912 | 1441493880 | 1442733032 |
|  | Q20 percentage | 96.24% | 96.68% | 95.79% | 97.95% | 97.91% | 97.99% |
|  | GC number | 1562792308 | 781730617 | 781061691 | 1486494935 | 743639204 | 742855731 |
|  | GC percentage | 50.58% | 50.60% | 50.56% | 50.48% | 50.51% | 50.46% |
|  | N number | 107304 | 37486 | 69818 | 13422 | 9921 | 3501 |
|  | N percentage | 0.00% | 0.00% | 0.00% | 0.00% | 0.00% | 0.00% |
